# Postprandial Effects of Breakfast Glycemic Index on Vascular Function among Young Healthy Adults: A Crossover Clinical Trial

**DOI:** 10.3390/nu9070712

**Published:** 2017-07-07

**Authors:** Natalia Sanchez-Aguadero, Maria C. Patino-Alonso, Sara Mora-Simon, Manuel A. Gomez-Marcos, Rosario Alonso-Dominguez, Benigna Sanchez-Salgado, Jose I. Recio-Rodriguez, Luis Garcia-Ortiz

**Affiliations:** 1 Primary Care Research Unit, The Alamedilla Health Center, Castilla and Leon Health Service (SACYL), Biomedical Research Institute of Salamanca (IBSAL), 37003 Salamanca, Spain; rosa90alonso@hotmail.com (R.A.-D.); benissanchez@gmail.com (B.S.-S.); 2 Primary Care Research Unit, The Alamedilla Health Center, Biomedical Research Institute of Salamanca (IBSAL), Department of Statistics, University of Salamanca, 37003 Salamanca, Spain; carpatino@usal.es; 3 Primary Care Research Unit, The Alamedilla Health Center, Biomedical Research Institute of Salamanca (IBSAL), School of Labor Relations of Zamora, University of Salamanca Affiliated Center, 37003 Salamanca, Spain; s_mora@usal.es; 4 Primary Care Research Unit, The Alamedilla Health Center, Castilla and Leon Health Service (SACYL), Biomedical Research Institute of Salamanca (IBSAL), Department of Medicine, University of Salamanca, 37003 Salamanca, Spain; magomez@usal.es; 5 Primary Care Research Unit, The Alamedilla Health Center, Castilla and Leon Health Service (SACYL), Biomedical Research Institute of Salamanca (IBSAL), Department of Nursing and Physiotherapy, University of Salamanca, 37003 Salamanca, Spain; donrecio@gmail.com; 6 Primary Care Research Unit, The Alamedilla Health Center, Castilla and Leon Health Service (SACYL), Biomedical Research Institute of Salamanca (IBSAL), Department of Biomedical and Diagnostic Sciences, University of Salamanca, 37003 Salamanca, Spain; lgarciao@usal.es

**Keywords:** breakfast, glycemic index, postprandial period, hemodynamics, vascular stiffness, blood glucose

## Abstract

This study aimed to evaluate the postprandial effects of high and low glycemic index (GI) breakfasts on vascular function. It was a crossover trial that included 40 young healthy adults (50% women), aged 20–40 years, who were recruited at primary care settings. They consumed three experimental breakfasts in randomized order, each one separated by a 1-week washout period: (1) control conditions (only water); (2) low GI (LGI) breakfast (29.4 GI and 1489 KJ energy); and (3) high GI (HGI) breakfast (64.0 GI and 1318 KJ energy). Blood samples were collected at 60 and 120 min after each breakfast to determine glucose and insulin levels. Vascular parameters were measured at 15 min intervals. Augmentation index (AIx) was studied as a primary outcome. Secondary outcomes comprised glucose, insulin, heart rate (HR) and pulse pressures (PPs). We found a trend toward increased AIx, HR and PPs for the HGI versus the LGI breakfast. A significant interaction between the type of breakfast consumed and all measured parameters was identified (*p* < 0.05) except for central PP. Stratifying data by sex, this interaction remained significant for AIx and augmentation pressure only in males (*p* < 0.05). In conclusion, breakfast GI could affect postprandial vascular responses in young healthy adults.

## 1. Introduction

Measures of vascular function are well-established indicators of cardiovascular health [1]. Central hemodynamics, central arterial stiffness, and indices of aortic wave reflection are more strongly related with cardiovascular risk than peripheral pressures [2,3,4,5]. However, there is no consensus regarding the nature of how acute changes in vascular parameters occur or how these changes associate with cardiovascular events [6,7]. Even though the postprandial state has been proposed as a contributor to the development of atherosclerosis [8], few studies have investigated the acute effects of food intake on vascular responses. Taylor et al. concluded that a liquid mixed meal acutely reduces peripheral and central pressures, aortic wave reflection, and arterial stiffness, all of which may be the result of an increase in insulin and/or visceral vasodilation [9]. There is accumulating evidence that postprandial hyperglycemia promotes endothelial dysfunction by inducing oxidative stress [10,11], whereas results from most studies suggest beneficial effects of insulin in endothelial function [12,13]. In fact, hyperinsulinemia appears to reduce the deleterious actions of hyperglycemia on vascular function [14]. Thus, interventions designed to reduce blood glucose such as those based on exercise may impact endothelial function and could have long-term consequences in vascular health [15,16].

Glycemic index (GI), which measures the speed with which a carbohydrate (CHO) containing-food is absorbed compared to a reference product (pure glucose) [17,18], is thought to be indicative of CHO quality. High GI diets are associated with increased cardiovascular disease (CVD) risks [19], while low GI diets could reduce CVD risks by decreasing postprandial glycemia with different favorable metabolic effects, including changes in insulin sensitivity, circulating serum lipid concentrations, and vascular function [20,21]. In a previous study, we observed an association between dietary GI and arterial stiffness in a sample of 1553 subjects who did not have CVD. Even with adjustments for multiple confounders, every increase of 5 GI units was significantly associated with a 0.11% increase in augmentation index (AIx) (*p* < 0.01) [22]. Although the postprandial glycemic responses associated with meals differing in CHO quantity and quality have been widely investigated [23,24,25], there are only a few studies that have examined its relationship with vascular parameters [26,27,28,29]. A study conducted in healthy elderly subjects reported no significant correlation between the glycemic effects of three 50 g CHO drinks with different GI and postprandial variations in blood pressure (BP) [27]. In turn, Greenfield et al. demonstrated that postprandial glucose and insulin changes in response to a meal’s CHO content resulted in varying AIx reductions in postmenopausal women [26].

It has been hypothesized that breakfast consumption and composition could affect cardiovascular risk (CVR) profile [30,31]. A trial aimed at comparing the effects of a high GI with a low GI breakfast replacement for three weeks in obese and overweight subjects reported decreased fasting glucose values after the low GI breakfast study period and no differences in BP nor insulin concentration between both interventions [32]. To date, little information is available regarding the acute effects of breakfast on vascular responses. In a sample population of healthy adults, Ahuja et al. found a significant reduction in peripheral pressures and central hemodynamics after breakfast consumption versus fasting (all differences *p* < 0.01), which might be attributed to splanchnic vasodilation [33]. Similarly, a high fat breakfast decreased central and peripheral BP in young healthy adults [34]. Despite the fact that traditional breakfast patterns usually are rich in CHO foods, the effects of distinct postprandial glycemic responses associated with breakfast GI on vascular parameters are not known. Therefore, the present study aimed to evaluate postprandial effects of low and high GI breakfasts on vascular function in a sample of young healthy Spanish adults.

## 2. Materials and Methods

### 2.1. Study Design and Population

A crossover clinical trial was conducted at The Alamedilla Primary Care Research Unit belonging to the Spanish Network for Preventive Activities and Health Promotion (REDIAPP) and the Biomedical Research Institute of Salamanca (IBSAL). The protocol for the Breakfast Glycemic Index (BGI) study (NCT02616276) has recently been published [35]. For the sample population, 40 subjects aged 20–40 years were selected through consecutive sampling at urban primary care health centers in Salamanca, Spain between 2015 and 2016. The exclusion criteria for the study included several factors: (1) history of cardiovascular events; (2) hypertension; (3) diabetes mellitus; (4) dyslipidemia; (5) pharmacological treatment for any of these conditions; (6) neurological and/or neuropsychological disorders; (7) consumption of toxic substances; (8) Celiac disease; (9) intolerance to lactose; (10) low-calorie and/or low-sodium diets; (11) pregnancy; and (12) any other circumstance that investigators think could interfere with the study procedures such as dietary conditions that can promote variability in measures (e.g., consumption of anti-oxidant or omega 3/6 supplements). The sample size was estimated accepting an alpha risk of 0.05 and a beta risk of 0.2 in a two-sided test. Forty subjects were considered enough to recognize a difference of ≥5 units in AIx as statistically significant, assuming a standard deviation of 10. A drop-out rate of 5% was anticipated. A flow diagram of the study can be seen in Supplemental Figure S1.

### 2.2. Intervention

Participants completed three interventions (control conditions and high and low GI breakfasts) in randomized order, each separated by a washout period of one week. Each visit lasted 2 h 40 min and occurred between 8:15 a.m. and 10:55 a.m.

#### Nutritional Composition of Each Intervention Arm

Nutritional composition of each intervention arm is detailed in Table 1.

1. Control conditions:
  This consisted of 350 mL of water served at room temperature.
2. High GI (HGI) breakfast:
  This consisted of 350 mL of water served at room temperature, 200 mL of grape juice (with 569 KJ/136 Kcal), 40 g of white bread (2 slices of 218 KJ/52 Kcal each) and 29 g of strawberry jam (with 313 KJ/75 Kcal) with an overall GI of 64.0.
3. Low GI (LGI) breakfast:
  This consisted of 350 mL of water served at room temperature, a 150 g apple (with 339 KJ/81 Kcal), 125 g low-fat natural yogurt (with 234 KJ/56 Kcal), 3 shelled walnuts (with 163 KJ/39 Kcal per unit), and 17.5 g of 72% dark chocolate (with 427 KJ/102 Kcal) with an overall GI of 29.4.

### 2.3. Study Protocol

Subjects were asked to fast for 12 h overnight prior to the study breakfast, to limit their physical activity, alcohol consumption, and smoking during the previous 24–48 h, and to maintain stable dietary habits between experimental trials.

On arrival at the research unit, they were weighed, and their height, waist, and hip circumferences were measured. Participants remained in a sitting position throughout the visit. After 5 min of rest, peripheral blood pressure was measured. Immediately after that, vascular parameters were obtained. Next, fasting blood samples were collected and vascular parameters were determined again. Subjects were provided with randomly assigned breakfasts to be consumed within 10 min. A timer was started at the beginning of the meal and additional measurements of vascular parameters were taken every 15 min. Furthermore, another two postprandial blood samples were taken at 60 and 120 min.

### 2.4. Outcomes

The primary outcome measure was change in AIx. Change in glucose, insulin, heart rate (HR) and pulse pressures were considered as secondary outcomes.

### 2.5. Variables and Measurement Instruments

#### 2.5.1. Vascular Function Evaluation Variables

The Mobil-O-Graph^®^ monitor [36] was used to measure peripheral and central BPs, pulse pressures (PPs), and central hemodynamics, including augmentation pressure (AP) and AIx standardized to a HR of 75 bpm. The monitor was set to obtain continuous measurements at −10, 0, 15–120 min (at 15 min intervals) with the subject sitting and resting his arm on a rigid surface. This device allows a noninvasive estimation of central BP (CBP) and AIx based on brachial pulse waves recorded with a conventional oscillometric BP cuff by applying a transfer function method. After determination of peripheral BP, the cuff instantly reinflates, and recordings for pulse waves are carried out at the diastolic blood pressure level (±5 mmHg) for approximately 10 s.

#### 2.5.2. Laboratory Variables

At the time of study entry and prior to the first intervention visit, fasting plasma creatinine, serum total cholesterol, HDL-cholesterol, LDL-cholesterol and triglycerides values were determined using standardized enzymatic automated methods.

During each study visit, three cannulated blood samples were collected at 0, 60, and 120 min in order to use these samples to measure serum glucose and insulin levels by ultraviolet-visible spectrophotometry and chemiluminescence, respectively. Serum was isolated by centrifugation and stored in a −20 °C freezer within 48–72 h until used for future analyses. Samples were treated and centrifuged by a single researcher under standard conditions. All analyses were performed in a laboratory complying with the external quality assurance programs of the Spanish Society of Clinical Chemistry and Molecular Pathology.

#### 2.5.3. Other Variables

Procedures for collecting sociodemographic and lifestyle-related data and obtaining anthropometric and peripheral blood pressure measurements have been reported in a prior publication [35].

### 2.6. Ethics

The Clinical Research Ethics Committee of the Health Area of Salamanca approved the study, and all participants gave written informed consent for the study according to the general recommendations of the Declaration of Helsinki. The trial was registered in ClinicalTrials.gov with identifier NCT02616276.

### 2.7. Statistics

Quantitative variables have been displayed as the mean ± standard deviation and qualitative variables have been expressed as frequencies and percentages. Student’s *t*-test for paired data was applied to compare repeated measures, using Bonferroni correction for multiple comparisons. Using analysis of variance (ANOVA) test, we compared the differences among the three types of breakfast. The Bonferroni test was used for post hoc analysis. To adequately evaluate the effect of the interventions, a repeated measures ANOVA has been performed using the general lineal model (GLM), stratifying by sex. The relationship of glycemia and insulinemia with vascular responses has been tested by Pearson’s correlation, using changes (mean differences) in outcome measures between baseline and 120 min after each breakfast. AIx incremental area under the curve (iAUC) was calculated using the trapezoidal method subtracting baseline values extrapolated over 30 min (0–30 min early phase and 60–120 min late phase) from the total AIx area. Analysis of variance (normally distributed data) was used to compare iAUC between breakfasts. Wilcoxon signed rank tests (skewed data) was used to compare results following the two phases of the postprandial period. The contrasting hypothesis established an alpha risk of 0.05 as the limit of statistical significance. The data have been analyzed using the IBM SPSS Statistics for Windows version 23.0 (IBM Corp, Armonk, NY, USA).

## 3. Results

The mean age of the participants was 28.1 years (50% women), of whom 3 (7.5%) were current smokers. Regarding physical activity, they performed a mean of 1973 METs/min/week. For diet quality index (DQI), the median score was 39.5 points. The BP was 106/66 mmHg and the HR was 70.2 bpm. All characteristics of the study subjects are presented in Table 2.

### 3.1. Changes in Central Hemodynamics in Response to Each Type of Breakfast

**Figure 1:**
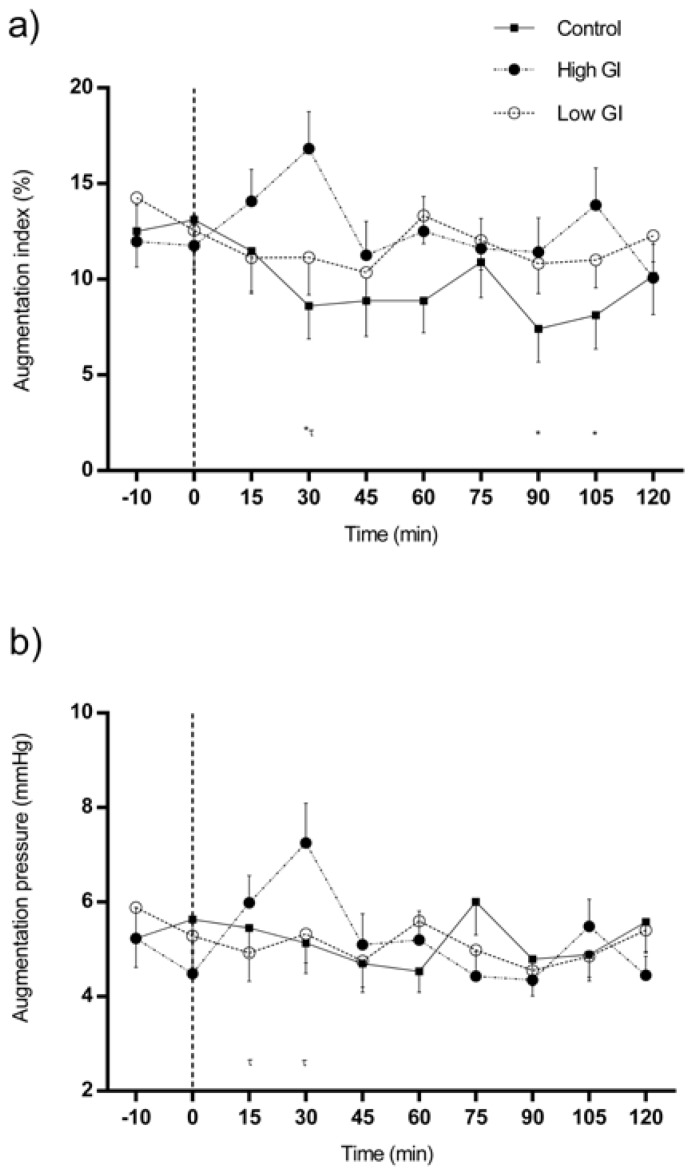
Changes in augmentation index (**a**) and augmentation pressure (**b**) in response to each type of breakfast (BF). All data were statistically analyzed with the Student’s *t*-test for paired data. Significant differences between baseline and individual time points of the same intervention: (**a**) Control conditions: 30 min (*p* < 0.05), 90 min (*p* = 0.01), 105 min (*p* = 0.016); High glycemic index (GI) BF: 30 min (*p* < 0.05); (**b**) High GI BF: 15 min (*p* = 0.012), 30 min (*p* < 0.01). * denotes significant changes (*p* < 0.05) in response to control conditions; and τ denotes significant changes (*p* < 0.05) in response to high GI BF.

Changes in central hemodynamics in response to each type of breakfast are shown in Figure 1. AIx was lower from 15 min after the ingestion of water and showed more prominent effects at 30, 90, and 105 min (all differences *p* < 0.05) (Figure 1a). Significant increments were detected in AIx (30 min) and AP (15–30 min) following intake of the HGI breakfast (all differences *p* < 0.05) (Figure 1a,b). No significant changes were observed in AIx or AP in response to the LGI breakfast (Figure 1a,b).

AIx responses were not significantly different between the early and late phase of the postprandial period for either breakfast.

Changes in central hemodynamics in response to each type of breakfast by sex can be seen in Supplemental Figure S2.

### 3.2. Changes in Heart Rate and Pulse Pressures in Response to Each Type of Breakfast

**Figure 2:**
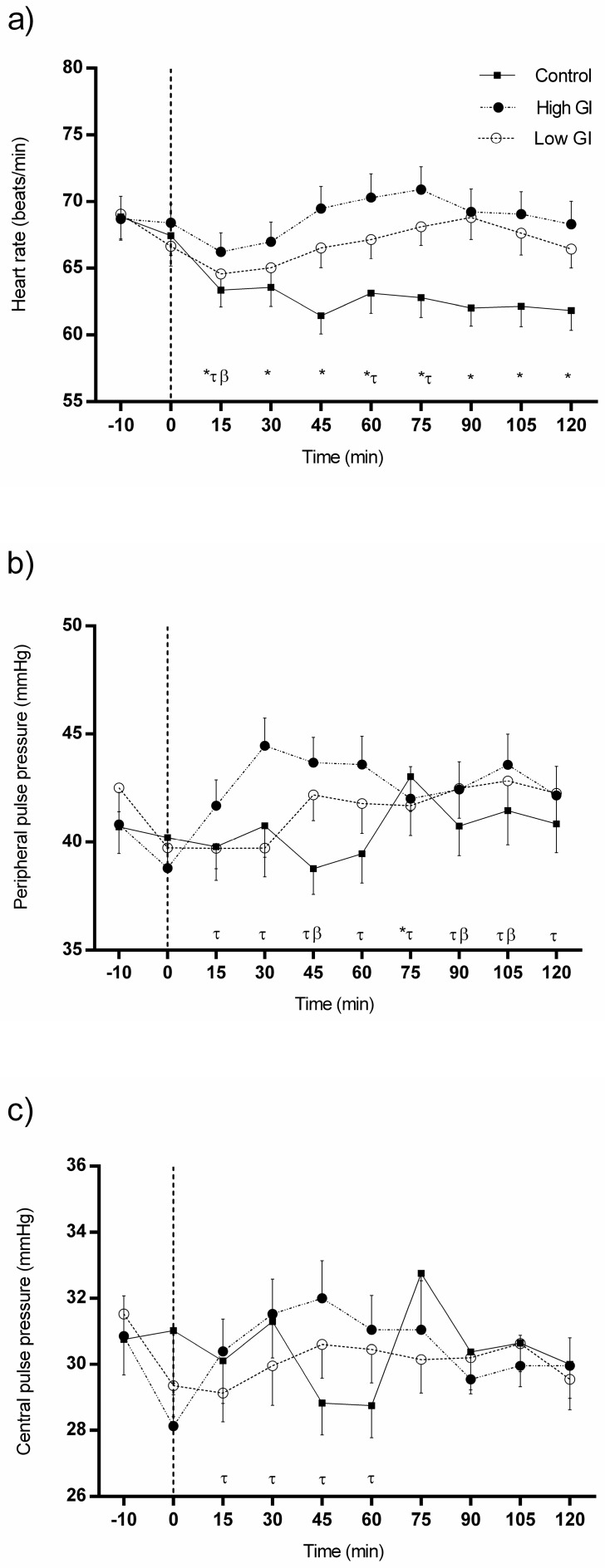
Changes in heart rate (**a**), peripheral pulse pressure (PPP) (**b**) and central pulse pressure (CPP) (**c**) in response to each type of breakfast (BF). All data were statistically analyzed with the Student’s *t*-test for paired data. Significant differences between baseline and individual time points of the same intervention: (**a**) Control conditions: 15–120 min (*p* < 0.001); High glycemic index (GI) BF: 15 min (*p* < 0.01), 60 min (*p* < 0.05), 75 min (*p* = 0.01); Low GI BF: 15 min (*p* = 0.013); (**b**) Control conditions: 75 min (*p* = 0.011); High GI BF: 15‒60, 90, and 105 min (*p* < 0.01), 75 and 120 min (*p* < 0.05); Low GI BF: 45, 90, and 105 min (*p* < 0.05). (c) High GI BF: 30 and 45 min (*p* < 0.01), 15 and 60 min (*p* < 0.05). * denotes significant changes (*p* < 0.05) in response to control conditions; τ denotes significant changes (*p* < 0.05) in response to high GI BF; and β denotes significant changes (*p* < 0.05) in response to low GI BF.

Changes in heart rate and pulse pressures in response to each type of breakfast are shown in Figure 2. HR (Figure 2a) was significantly lower than baseline at all time points following water ingestion (all differences *p* < 0.001). In contrast, HR showed significantly higher values between 60 and 75 min (70.30 ± 11.19 versus 70.90 ± 10.81) after the HGI breakfast in comparison with baseline (68.40 ± 8.85; all measurements *p* < 0.05). Likewise, a similar tendency toward an increase in HR was observed in response to the LGI breakfast.

On the other hand, water intake did not elicit a significant change in peripheral pulse pressure (PPP) or central pulse pressure (CPP) (Figure 2b,c). Nonetheless, PPP was higher than at baseline during the 120 min period after consumption of the HGI breakfast (all differences *p* < 0.05) (Figure 2b), and CPP was also significantly greater from 15 to 60 min postprandially (all measurements *p* < 0.05) (Figure 2c). Similarly, in response to the LGI breakfast, PPP was significantly higher than baseline (29.35 ± 5.87) at 45, 90, and 105 min (30.60 ± 6.47 versus 30.20 ± 7.00 versus 30.63 ± 5.46; all differences *p* < 0.05) (Figure 2b). Differences in CPP were similar (Figure 2c).

Changes in heart rate and pulse pressures in response to each type of breakfast by sex can be seen in Supplemental Figure S3.

### 3.3. Changes in Glucose and Insulin in Response to Each Type of Breakfast

**Figure 3:**
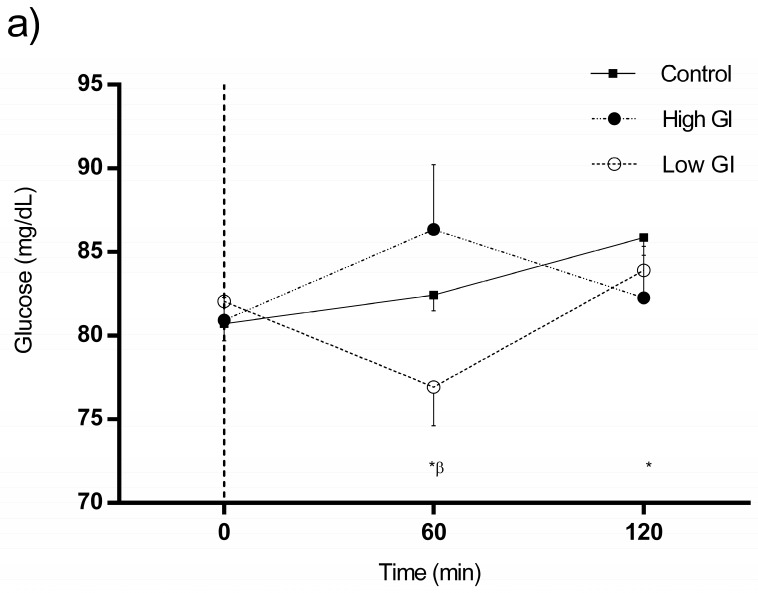

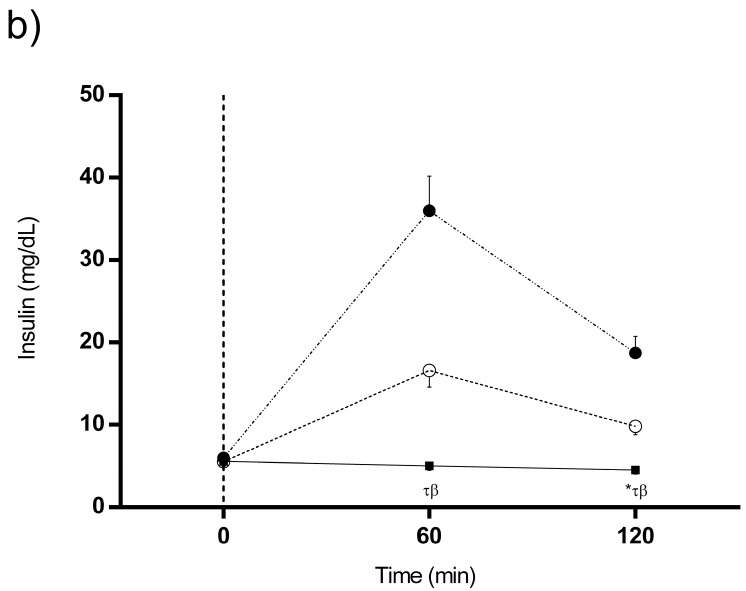
Changes in serum glucose (**a**) and insulin (**b**) in response to each type of breakfast (BF). All data were statistically analyzed with the Student’s *t*-test for paired data. Significant differences between baseline and individual time points of the same intervention: (**a**) Control conditions: 60 and 120 min (*p* < 0.01); Low GI BF: 60 min (*p* = 0.026); (**b**) Control conditions: 120 min (*p* = 0.011); High GI BF: 60 and 120 min (*p* < 0.001); Low GI BF: 60 and 120 min (*p* < 0.001). * denotes significant changes (*p* < 0.05) in response to control conditions; τ denotes significant changes (*p* < 0.05) in response to high GI BF; and β denotes significant changes (*p* < 0.05) in response to low GI BF.

Changes in glucose and insulin in response to each type of breakfast are shown in Figure 3. After the ingestion of water, glucose was elevated at 60 and 120 min (82.43 ± 5.99 versus 85.88 ± 6.80) in comparison with baseline (80.73 ± 6.54; all measurements *p* ≤ 0.001) (Figure 3a), whereas insulin levels were reduced, especially at 120 min (5.55 ± 3.68 versus 4.54 ± 3.31; *p* = 0.011) (Figure 3b). In turn, glucose and insulin had both increased at 60 min following the HGI breakfast (Figure 3a,b), although the changes observed in glucose were not significantly different from baseline (*p* = 0.169) (Figure 3a). Insulin remained elevated 120 min after the HGI breakfast (5.96 ± 4.54 versus 18.72 ± 12.76; all differences *p* < 0.001) (Figure 3b). Finally, in response to the LGI breakfast, glucose had decreased at 60 min (76.93 ± 14.65) compared with baseline (82.05 ± 7.36; *p* = 0.026) (Figure 3a). Insulin, however, had increased 60 and 120 min after consumption of the LGI breakfast, with the maximum effect occurring at 60 min (5.51 ± 3.77 versus 16.60 ± 12.58; all measurements *p* < 0.001) (Figure 3b).

Changes in glucose and insulin in response to each type of breakfast by sex can be seen in Supplemental Figure S4.

### 3.4. Comparisons between Postprandial Responses to Each Type of Breakfast

Table 3 shows the comparisons between postprandial responses induced by each experimental breakfast. There were no significant differences in any of the baseline parameters. AIx, AP, HR and CPP showed no differences between either breakfasts (HGI versus LGI breakfast). PPP at 30 min was lower for the LGI breakfast than the HGI breakfast (mean difference: −4.73 mmHg; *p* = 0.047). Glucose values at 60 min after consumption of the HGI breakfast were significantly higher than those after the LGI breakfast (mean difference: +9.43 mg/dL; *p* = 0.041). Postprandial insulin concentrations over 120 min were greater when subjects consumed the HGI breakfast compared to the LGI breakfast (mean difference: +19.38 mg/dL and +8.92 mg/dL at 60 and 120 min, respectively; all differences *p* < 0.001).

There were no significant differences between breakfasts in the AIx increase early phase (iAUC, *p* = 0.502) nor late phase (iAUC, *p* = 0.254).

Comparisons between postprandial responses to each type of breakfast by sex can be seen in Supplemental Tables S1 and S2.

### 3.5. Effects of Type of Breakfast on Postprandial Responses

Repeated measures ANOVA showed a significant interaction between the type of breakfast and all measured parameters (all differences *p* < 0.05), with the exception of CPP (*p* = 0.079). When data were stratified by sex, this interaction remained significant for AIx (*p* < 0.01) and AP (*p* < 0.05) only in males, glucose (*p* < 0.05) only in females, and insulin (*p* < 0.001) and HR (*p* < 0.05) in both sexes. In contrast, significance was lost for PPP in both sexes. More details about the analysis performed can be seen in Supplemental Table S3.

Correlations run between changes from baseline to 120 min in glucose, insulin and vascular parameters revealed that change in insulin was inversely associated with change in HR *(r* = −0.344, *p* < 0.05) during control conditions test. We also found negative associations between change in insulin and changes in PPP (*r* = −0.362, *p* < 0.05) and CPP (*r* = −0.364, *p* < 0.05) during LGI breakfast test. Changes in glucose and insulin did not correlate with changes in vascular parameters during HGI breakfast test.

## 4. Discussion

Breakfast GI influenced the evolution of most of the observed postprandial responses. Although we found no significant differences in vascular parameters when changes in each one of them after consumption of the HGI and LGI breakfasts were compared, it appears that the HGI breakfast led to a more intense response on AIx, HR, PPs, and blood glucose and insulin concentrations. It could be inferred from these data that sympathetic nervous system activity was potentially greater and, in turn, related to elevated arterial stiffness. These results suggest that the LGI breakfast could provide more favorable acute vascular responses than the HGI breakfast in young healthy adults. Inverse correlations found between changes in insulin and pulse pressures during LGI breakfast test could indicate a contribution of insulinemia in the differing vascular responses elicited by each breakfast. In this sense, Greenfield et al. [26] demonstrated that the magnitude of the insulin response induced by the carbohydrate content of a meal was an important determinant of postprandial arterial stiffness.

In conjunction with the current study, some authors have demonstrated increased arterial stiffness or impaired endothelial function under postprandial conditions in overweight or obese adults and those with metabolic syndrome and/or type 2 diabetes [28,37,38]. Given that humans spend most of the day in the postprandial state, which has been identified as proatherogenic [8], our findings might have clinical relevance in relation to cardiovascular risks because they could provide necessary information for novel dietary approaches in order to help reduce postprandial arterial stiffness and endothelial dysfunction.

There are just a few studies investigating the effects on vascular function of breakfasts differing in nutrient composition. A trial by Ahuja et al. reported a significantly lower brachial and central BP, CPP, AP, and AIx and a higher HR after a light breakfast (1301 KJ energy) than after the ingestion of water alone (all differences *p* < 0.01) [33]. Likewise, reductions in central systolic, diastolic and peripheral diastolic BP were observed after the consumption of a breakfast high in saturated fats (450 Kcal energy) (all measurements *p* < 0.05), while PPP, CPP, HR, and pulse wave velocity (PWV) did not differ significantly [34]. A possible explanation for the discrepancy in our results may be related to the variety of methods used to assess vascular parameters and differences in macronutrient content of experimental meals.

To our knowledge, this study shows for the first time that breakfasts varying in GI can affect vascular parameters differently. The mechanisms by which postprandial vascular responses occur are unclear. Although hyperglycemia is considered detrimental in many cases, in our study people were fairly normoglycemic, even with the HGI breakfast. Thus, acute changes in vascular parameters described could be mediated by factors other than glycemic responses such as increased sympathetic nerve activity and peripheral resistance that occur postprandially [39].

In our study, there are several experimental considerations that should be highlighted. Firstly, the inability to detect significant differences in postprandial vascular responses could be due to the participants being young healthy adults, so there may be no improvement to observe since they are healthy. Secondly, our results show that AIx and AP responses are affected by the type of breakfast only in males. This could be related to physiologic differences such as greater capillary density as well as oxidative metabolism for women compared with men in this age group. Previous studies have already reported gender differences in postprandial vascular responses [40,41]. However, in the present study, sex specific effects might potentially be masked by the variability that could have introduced in outcome measures the lack of control for menstrual cycle in female subjects [42]. Thirdly, we cannot exclude the possible contribution of other nutritional components on the acute effects of breakfasts, because they differ in volume, energy and macronutrient distribution. In this regard, conditions associated with a slowing gastric emptying such as volume, protein, fiber or fat are known to attenuate the magnitude of postprandial vascular responses [27]. Besides GI, different quantities of CHO served in breakfasts may have influenced vascular responsiveness. Fourthly, we did not standardize food intake prior to experimental trials, so an impact of variations in hormone and metabolic responses that occur with a second meal [43] cannot be excluded. Finally, the experimental breakfasts could induce vascular changes later than 120 min.

## 5. Conclusions

Breakfast GI could affect vascular parameters in young healthy adults. Postprandial vascular responses following the HGI and LGI breakfasts were similar, although the HGI breakfast appears to have more intense effects on AIx, HR and PPs. This suggests that the modifications in breakfast GI may be a potentially useful dietary alternative that could benefit vascular function in the postprandial state. Nevertheless, our findings are delimited to breakfast meals, so further research is required to clarify whether the influence of breakfast GI on vascular parameters would hold over the day or chronically over weeks.

## Figures and Tables

**Table 1:** Nutritional composition of each breakfast.

Type of Breakfast	GI, %	Energy, Kcal/KJ	Carbohydrates, g (%)	Protein, g (%)	Fat, g (%)	Saturated Fat ^a^, g	Fiber, g (%)	Soluble Fiber ^b^, g
Control conditions	-	-	-	-	-	-	-	-
High GI	64.0	315/1318	72.0 (91.4)	3.9 (5.0)	0.9 (2.6)	0.29	1.6 (1.0)	0.36
Low GI	29.4	356/1489	31.5 (35.4)	9.7 (10.9)	19.9 (50.3)	1.54	6.0 (3.4)	1.57

^a^ Saturated fat suppose 32% and 8% of fats consumed in high and low GI breakfasts, respectively; ^b^ Soluble fiber suppose 23% and 26% of fiber consumed in high and low GI breakfasts, respectively. GI: Glycemic index.

**Table 2:** Baseline characteristics of the study population.

	Global (*N* = 40) Mean or n/SD or %	Males (*n* = 20) Mean or n/SD or %	Females (*n* = 20) Mean or n/SD or %
Age (years)	28.1 (6.3)	28.7 (6.8)	27.4 (5.9)
Diet Quality Index, DQI (total score)	39.5 (2.8)	38.4 (2.6)	40.6 (2.5)
Physical Activity (METS/min/week)	1973.1 (1239.5)	2448.0 (1290.4)	1498.2 (1006.8)
Current smoker (*n*, %)	3 (7.5)	2 (10.0)	1 (5.0)
Alcohol consumption (g/week)	40.8 (48.1)	52.5 (55.9)	29.0 (36.5)
Systolic blood pressure (mmHg)	106.4 (9.8)	112.0 (7.5)	100.7 (8.5)
Diastolic blood pressure (mmHg)	66.2 (6.9)	67.1 (6.9)	65.4 (6.9)
Heart rate (bpm)	70.2 (12.5)	65.7 (11.3)	74.8 (12.2)
Body mass index (kg/m^2^)	23.5 (3.6)	24.5 (3.1)	22.5 (3.9)
Waist circumference (cm)	78.8 (10.1)	84.4 (8.5)	73.2 (8.4)
Hip circumference (cm)	102.2 (7.5)	103.9 (7.1)	100.4 (7.7)
Total cholesterol (mg/dL)	169.2 (29.4)	168.7 (30.4)	169.8 (29.0)
HDL-cholesterol (mg/dL)	61.9 (15.3)	54.5 (12.2)	69.3 (14.8)
LDL-cholesterol (mg/dL)	92.5 (27.5)	97.9 (28.6)	87.1 (25.9)
Triglycerides (mg/dL)	74.3 (31.2)	81.3 (33.6)	67.2 (27.6)
Creatinine (mg/dL)	0.76 (0.19)	0.88 (0.17)	0.64 (0.12)

Data for qualitative variables are expressed as *n* (%) and quantitative variables as mean ± standard deviation.

**Table 3:** Comparisons between postprandial responses to each type of breakfast.

**Augmentation index (AIx), %**	**0 min-AIx**			**30 min-AIx**			**60 min-AIx**			**120 min-AIx**		
	**Mean**	**SD**	***p***	**Mean**	**SD**	***p***	**Mean**	**SD**	***p***	**Mean**	**SD**	***p***
Control conditions	13.10	10.67	0.864	8.60	10.87	0.008	8.88	10.60	0.139	10.18	12.75	0.599
HGI breakfast	11.75	9.96		16.83 ^¥^	12.20		12.50	11.60		10.08	11.07	
LGI breakfast	12.55	12.81		11.15	12.42		13.33	9.38		12.28	8.65	
**Augmentation pressure (AP), mmHg**	**0 min-AP**			**30 min-AP**			**60 min-AP**			**120 min-AP**		
	**Mean**	**SD**	***p***	**Mean**	**SD**	***p***	**Mean**	**SD**	***p***	**Mean**	**SD**	***p***
Control conditions	5.63	4.49	0.417	5.13	4.10	0.069	4.53	2.81	0.355	5.58	4.22	0.265
HGI breakfast	4.48	3.11		7.25	5.32		5.20	3.92		4.45	2.58	
LGI breakfast	5.28	4.18		5.33	3.90		5.60	3.26		5.40	2.87	
**Heart rate (HR), bpm**	**0 min-HR**			**30 min-HR**			**60 min-HR**			**120 min-HR**		
	**Mean**	**SD**	***p***	**Mean**	**SD**	***p***	**Mean**	**SD**	***p***	**Mean**	**SD**	***p***
Control conditions	67.45	9.28	0.696	63.58	8.94	0.249	63.13	9.50	0.007	61.85	9.50	0.013
HGI breakfast	68.40	8.85		66.98	9.36		70.30 ^¥^	11.19		68.30 ^¥^	10.86	
LGI breakfast	66.65	9.43		65.03	9.01		67.15	9.04		66.45	9.16	
**Peripheral pulse pressure (PPP), mmHg**	**0 min-PPP**			**30 min-PPP**			**60 min-PPP**			**120 min-PPP**		
	**Mean**	**SD**	***p***	**Mean**	**SD**	***p***	**Mean**	**SD**	***p***	**Mean**	**SD**	***p***
Control conditions	40.20	8.57	0.678	40.75	9.26	0.040	39.45	8.50	0.094	40.85	8.50	0.721
HGI breakfast	38.80	7.01		44.45 ^#^	8.17		43.60	8.19		42.15	8.60	
LGI breakfast	39.73	5.76		39.73	8.43		41.78	8.68		42.25	8.78	
**Central pulse pressure (CPP), mmHg**	**0 min-CPP**			**30 min-CPP**			**60 min-CPP**			**120 min-CPP**		
	**Mean**	**SD**	***p***	**Mean**	**SD**	***p***	**Mean**	**SD**	***p***	**Mean**	**SD**	***p***
Control conditions	31.03	10.05	0.232	31.30	6.91	0.558	28.75	6.13	0.252	30.00	6.53	0.912
HGI breakfast	28.13	6.03		31.53	6.70		31.05	6.60		29.48	5.35	
LGI breakfast	29.35	5.87		29.95	7.49		30.45	6.41		29.55	5.84	
**Glucose, mg/dL**	**0 min-Glucose**						**60 min-Glucose**			**120 min-Glucose**		
	**Mean**	**SD**	***p***				**Mean**	**SD**	***p***	**Mean**	**SD**	***p***
Control conditions	80.73	6.54	0.702				82.43	5.99	0.045	85.88	6.80	0.462
HGI breakfast	80.93	8.70					86.35^#^	24.43		82.28	19.46	
LGI breakfast	82.05	7.36					76.93	14.65		83.90	8.76	
**Insulin, mg/dL**	**0 min-Insulin**						**60 min-Insulin**			**120 min-Insulin**		
	**Mean**	**SD**	***p***				**Mean**	**SD**	***p***	**Mean**	**SD**	***p***
Control conditions	5.55	3.68	0.858				5.00	3.44	<0.001	4.54	3.31	<0.001
HGI breakfast	5.96	4.54					35.98 ^¥ #^	26.71		18.72 ^¥ #^	12.76	
LGI breakfast	5.51	3.77					16.60 ^¥^	12.58		9.80 ^¥^	6.31	

ANOVA test has been used. Post-hoc contrasts were performed by Bonferroni test. ^¥^ Significantly different (*p* < 0.05) from control conditions. ^#^ Significant difference (*p* < 0.05) between HGI and LGI breakfasts. HGI: High glycemic index; LGI: Low glycemic index.

## Supplementary Materials

The following are available online at www.mdpi.com/2072-6643/9/7/712/s1, Figure S1: Flow-chart of the Breakfast Glycemic Index (BGI) study, Figure S2: Changes in central hemodynamics in response to each type of breakfast by sex, Figure S3: Changes in heart rate and pulse pressures in response to each type of breakfast by sex, Figure S4: Changes in glucose and insulin in response to each type of breakfast by sex, Table S1: Comparisons between postprandial responses to each type of breakfast in males, Table S2: Comparisons between postprandial responses to each type of breakfast in females, Table S3: Effects of type of breakfast on postprandial responses.
