# Supplementary material for: Postprandial Effects of Breakfast Glycemic Index on Vascular Function among Young Healthy Adults: A Crossover Clinical Trial
Source: Nutrients. 2017 Jul 7;9(7):712. doi: 10.3390/nu9070712 (PMC5537827; doi:10.3390/nu9070712)
Supplement: Supplementary file 1 [file nutrients-09-00712-s001.zip › Supplemental figures S2-S4.pdf]

Supplemental figures

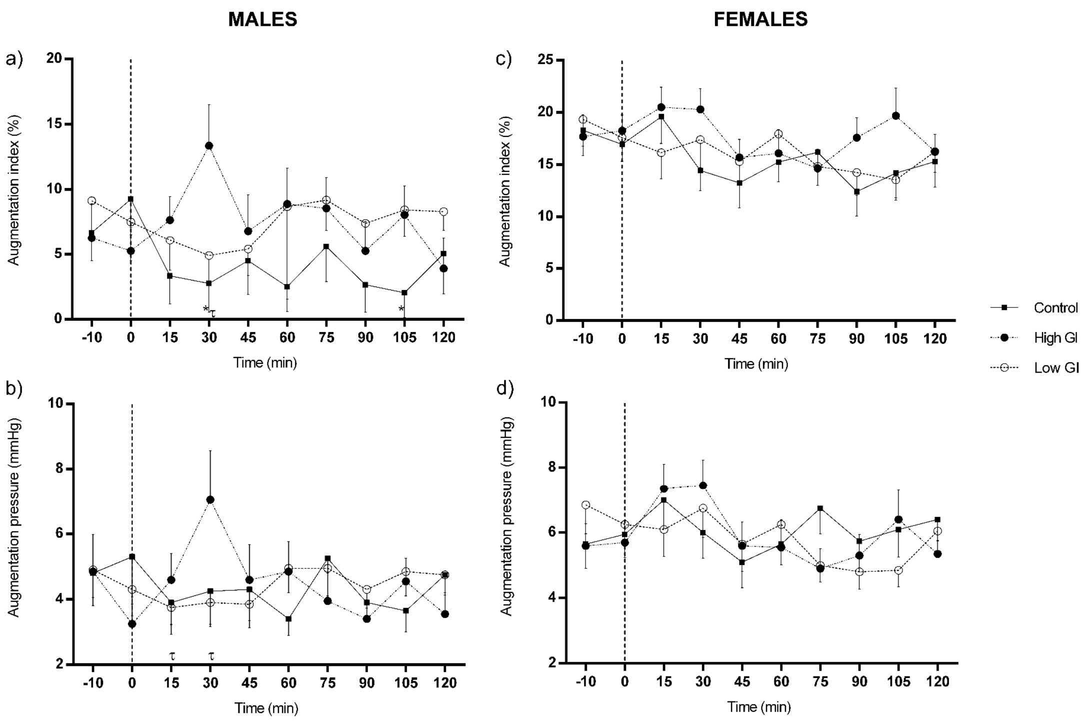

Figure S2. Changes in central hemodynamics in response to each type of breakfast (BF) by sex. All data were statistically analyzed with the Student's t-test for paired data. * denotes significant changes (p<0.05) in response to control conditions; τ denotes significant changes (p<0.05) in response to high GI BF; and β denotes significant changes (p<0.05) in response to low GI BF.

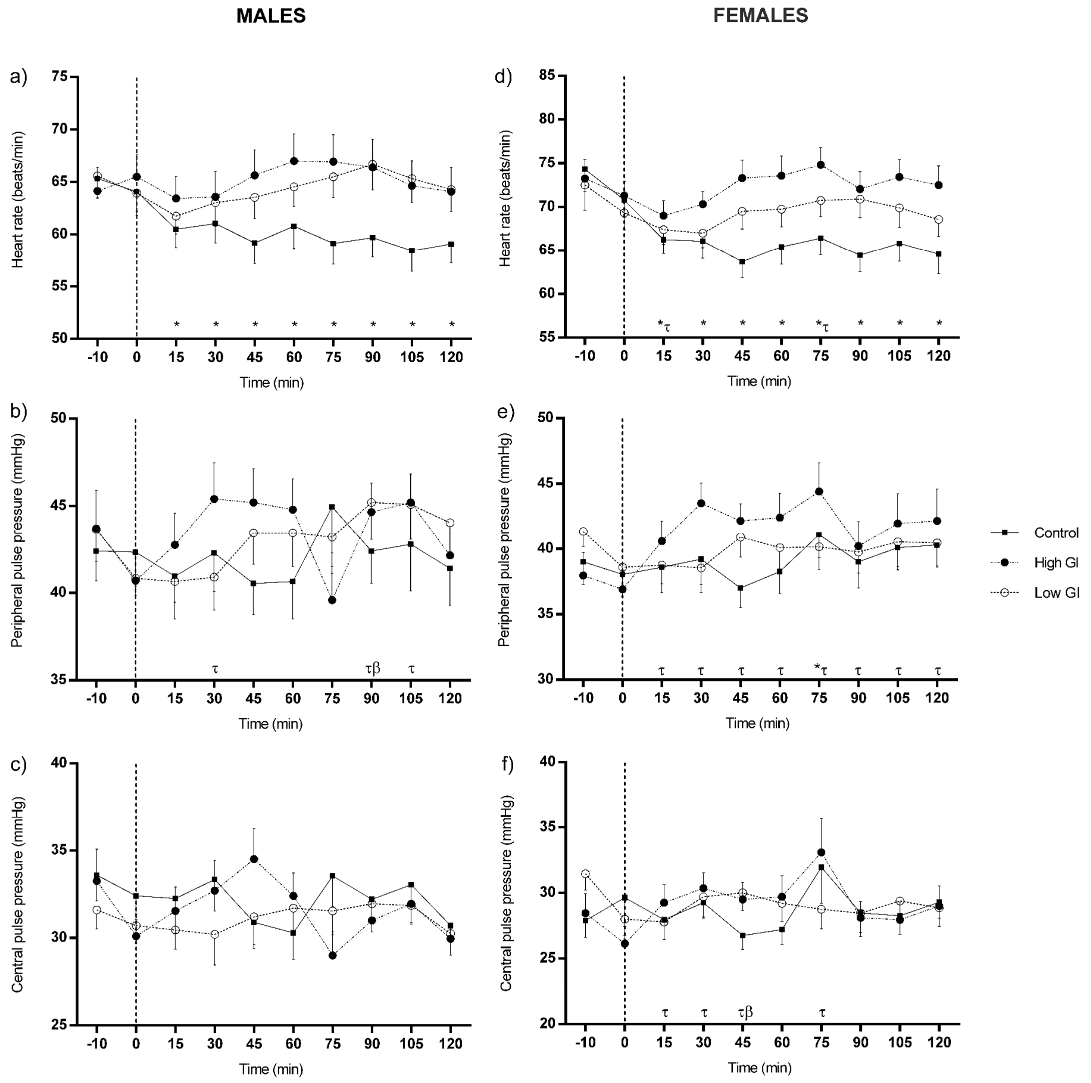

Figure S3. Changes in heart rate and pulse pressures in response to each type of breakfast (BF) by sex. All data were statistically analyzed with the Student's t-test for paired data. * denotes significant changes ($p<0.05$) in response to control conditions; τ denotes significant changes ($p<0.05$) in response to high GI BF; and β denotes significant changes ($p<0.05$) in response to low GI BF.

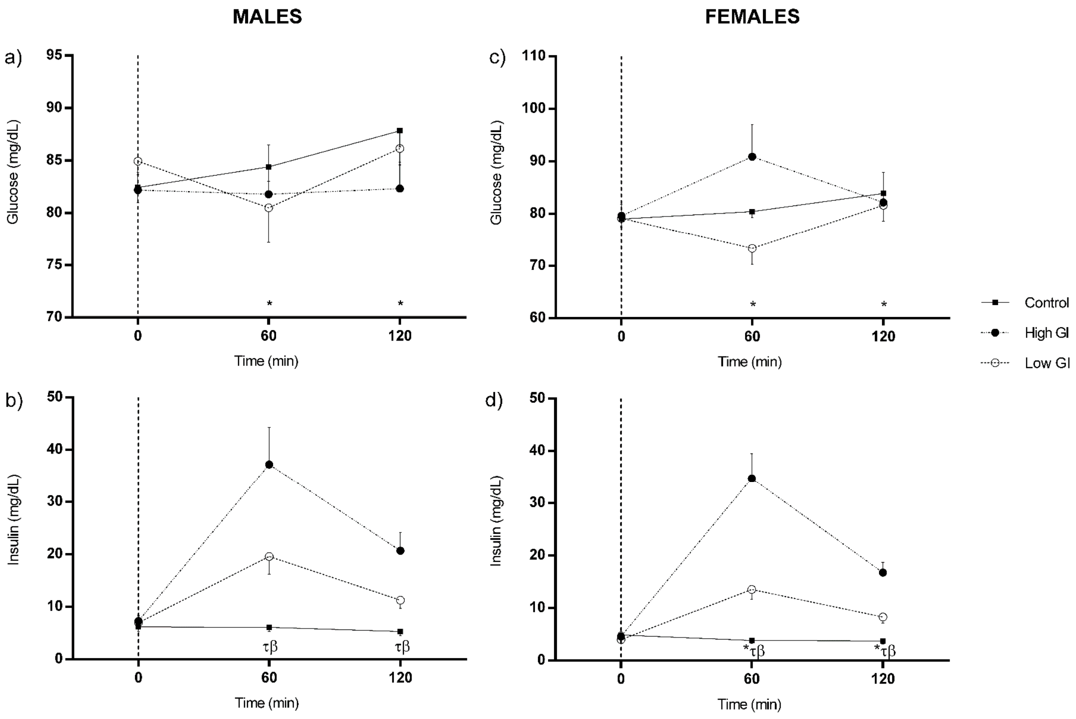

Figure S4. Changes in glucose and insulin in response to each type of breakfast (BF) by sex. All data were statistically analyzed with the Student's t-test for paired data. * denotes significant changes ($p<0.05$) in response to control conditions; τ denotes significant changes ($p<0.05$) in response to high GI BF; and β denotes significant changes ($p<0.05$) in response to low GI BF.
