# Supplementary material for: Postprandial Effects of Breakfast Glycemic Index on Vascular Function among Young Healthy Adults: A Crossover Clinical Trial
Source: Nutrients. 2017 Jul 7;9(7):712. doi: 10.3390/nu9070712 (PMC5537827; doi:10.3390/nu9070712)
Supplement: Supplementary file 1 [file nutrients-09-00712-s001.zip › Table S1.pdf]

Table S1: Comparisons between postprandial responses to each type of breakfast in males

**AUGMENTATION INDEX (AIx), %**	**0 min- AIx**			**30 min- AIx**			**60 min- AIx**			**120 min- AIx**		
	**Mean**	**SD**	**p**	**Mean**	**SD**	**p**	**Mean**	**SD**	**p**	**Mean**	**SD**	**p**
Control conditions	9.25	12.17		2.75	9.82		2.50	8.56		5.05	13.93	
HGI breakfast	5.25	8.27	0.549	13.35[¥]	14.14	0.011	8.90	12.18	0.063	3.90	10.71	0.416
LGI breakfast	7.50	13.47		4.90	9.54		8.70	7.17		8.30	6.46	
**AUGMENTATION PRESSURE (AP), mmHg**	**0 min-AP**			**30 min-AP**			**60 min-AP**			**120 min-AP**		
	**Mean**	**SD**	**p**	**Mean**	**SD**	**p**	**Mean**	**SD**	**p**	**Mean**	**SD**	**p**
Control conditions	5.30	5.54		4.25	4.53		3.40	2.28		4.75	5.15	
HGI breakfast	3.25	2.69	0.299	7.05	6.78	0.107	4.85	4.11	0.265	3.55	2.91	0.514
LGI breakfast	4.30	3.63		3.90	3.28		4.95	3.33		4.75	2.79	
**HEART RATE (HR), bpm**	**0 min-HR**			**30 min-HR**			**60 min-HR**			**120 min-HR**		
	**Mean**	**SD**	**p**	**Mean**	**SD**	**p**	**Mean**	**SD**	**p**	**Mean**	**SD**	**p**
Control conditions	64.10	8.63		61.05	8.38		60.80	9.66		59.10	8.25	
HGI breakfast	65.50	9.51	0.843	63.60	10.79	0.689	67.00	11.65	0.151	64.10	10.36	0.147
LGI breakfast	63.95	9.54		63.05	10.06		64.55	8.41		64.30	9.37	
**PERIPHERAL PULSE PRESSURE (PPP), mmHg**	**0 min-PPP**			**30 min-PPP**			**60 min-PPP**			**120 min-PPP**		
	**Mean**	**SD**	**p**	**Mean**	**SD**	**p**	**Mean**	**SD**	**p**	**Mean**	**SD**	**p**
Control conditions	42.35	8.96		42.30	9.86		40.65	9.55		41.40	9.44	
HGI breakfast	40.70	7.18	0.720	45.40	9.32	0.294	44.80	7.95	0.316	42.15	5.72	0.589
LGI breakfast	40.85	4.38		40.90	8.37		43.45	8.63		44.05	9.36	

CENTRAL PULSE PRESSURE (CPP), mmHg	0 min-CPP			30 min-CPP			60 min-CPP			120 min-CPP		
	**Mean**	**SD**	**p**	**Mean**	**SD**	**p**	**Mean**	**SD**	**p**	**Mean**	**SD**	**p**
Control conditions	32.40	5.98	0.390	33.35	8.11	0.418	30.30	6.88	0.573	30.70	7.52	0.916
HGI breakfast	30.10	5.66		32.70	7.78		32.40	5.82		29.95	3.27	
LGI breakfast	30.70	4.62		30.20	7.83		31.70	6.41		30.25	5.51	
**GLUCOSE, mg/dL**	**0 min-Glucose**						**60 min-Glucose**			**120 min-Glucose**		
	**Mean**	**SD**	**p**				**Mean**	**SD**	**p**	**Mean**	**SD**	**p**
Control conditions	82.45	6.67	0.366				84.40	6.03	0.715	87.85	6.89	0.125
HGI breakfast	82.20	7.53					81.80	21.03		82.35	11.17	
LGI breakfast	84.95	5.87					80.50	14.88		86.15	6.95	
**INSULIN, mg/dL**	**0 min-Insulin**						**60 min-Insulin**			**120 min-Insulin**		
	**Mean**	**SD**	**p**				**Mean**	**SD**	**p**	**Mean**	**SD**	**p**
Control conditions	6.21	4.16	0.703				6.13	3.88	<0.001	5.32	3.82	<0.001
HGI breakfast	7.30	4.54					37.21[¥#]	31.86		20.68[¥#]	15.79	
LGI breakfast	6.99	3.99					19.62	15.31		11.29	7.21	

ANOVA test has been used. Post-hoc contrasts were performed by Bonferroni test.

[¥] Significantly different ($p < 0.05$) from control conditions.

[#] Significant difference ($p < 0.05$) between HGI and LGI breakfasts.

HGI: High glycemic index; LGI: Low glycemic index.
