# Supplementary material for: Postprandial Effects of Breakfast Glycemic Index on Vascular Function among Young Healthy Adults: A Crossover Clinical Trial
Source: Nutrients. 2017 Jul 7;9(7):712. doi: 10.3390/nu9070712 (PMC5537827; doi:10.3390/nu9070712)
Supplement: Supplementary file 1 [file nutrients-09-00712-s001.zip › Table S2.pdf]

Table S2: Comparisons between postprandial responses to each type of breakfast in females

AUGMENTATION INDEX (AIx), %	0 min- AIx			30 min- AIx			60 min- AIx			120 min- AIx		
	Mean	SD	p	Mean	SD	p	Mean	SD	p	Mean	SD	p
Control conditions	16.95	7.37	0.882	14.45	8.61	0.187	15.25	8.48	0.643	15.30	9.19	0.921
HGI breakfast	18.25	6.79		20.30	8.94		16.10	10.03		16.25	7.53	
LGI breakfast	17.60	10.08		17.40	11.98		17.95	9.17		16.25	8.87	
**AUGMENTATION PRESSURE (AP), mmHg**	**0 min-AP**			**30 min-AP**			**60 min-AP**			**120 min-AP**		
	**Mean**	**SD**	**p**	**Mean**	**SD**	**p**	**Mean**	**SD**	**p**	**Mean**	**SD**	**p**
Control conditions	5.95	3.25	0.894	6.00	3.52	0.466	5.65	2.89	0.769	6.40	2.95	0.437
HGI breakfast	5.70	3.06		7.45	3.49		5.55	3.79		5.35	1.87	
LGI breakfast	6.25	4.55		6.75	4.03		6.25	3.14		6.05	2.87	
**HEART RATE (HR), bpm**	**0 min-HR**			**30 min-HR**			**60 min-HR**			**120 min-HR**		
	**Mean**	**SD**	**p**	**Mean**	**SD**	**p**	**Mean**	**SD**	**p**	**Mean**	**SD**	**p**
Control conditions	70.80	8.87	0.744	66.10	8.96	0.191	65.45	8.98	0.028	64.60	10.06	0.040
HGI breakfast	71.30	7.25		70.35	6.28		73.60[¥]	9.91		72.50[¥]	9.89	
LGI breakfast	69.35	8.73		67.00	7.56		69.75	9.10		68.60	8.65	
**PERIPHERAL PULSE PRESSURE (PPP), mmHg**	**0 min-PPP**			**30 min-PPP**			**60 min-PPP**			**120 min-PPP**		
	**Mean**	**SD**	**p**	**Mean**	**SD**	**p**	**Mean**	**SD**	**p**	**Mean**	**SD**	**p**
Control conditions	38.05	7.80	0.739	39.20	8.58	0.117	38.25	7.36	0.281	40.30	7.66	0.770
HGI breakfast	36.90	6.46		43.50	6.96		42.40	8.44		42.15	10.92	
LGI breakfast	38.60	6.79		38.55	8.53		40.10	8.63		40.45	7.98	

CENTRAL PULSE PRESSURE (CPP), mmHg	0 min-CPP			30 min-CPP			60 min-CPP			120 min-CPP		
	**Mean**	**SD**	**p**	**Mean**	**SD**	**p**	**Mean**	**SD**	**p**	**Mean**	**SD**	**p**
Control conditions	29.65	12.95	0.480	29.25	4.84	0.841	27.20	4.98	0.412	29.30	5.48	0.973
HGI breakfast	26.15	5.86		30.35	5.34		29.70	7.20		29.00	6.90	
LGI breakfast	28.00	6.75		29.70	7.34		29.20	6.33		28.85	6.23	
**GLUCOSE, mg/dL**	**0 min-Glucose**						**60 min-Glucose**			**120 min-Glucose**		
	**Mean**	**SD**	**p**				**Mean**	**SD**	**p**	**Mean**	**SD**	**p**
Control conditions	79.00	6.10	0.964				80.45	5.39	0.011	83.90	6.26	0.901
HGI breakfast	79.65	9.76					90.90[#]	27.19		82.20	25.55	
LGI breakfast	79.15	7.69					73.35	13.88		81.65	9.93	
**INSULIN, mg/dL**	**0 min-Insulin**						**60 min-Insulin**			**120 min-Insulin**		
	**Mean**	**SD**	**p**				**Mean**	**SD**	**p**	**Mean**	**SD**	**p**
Control conditions	4.89	3.09	0.727				3.88	2.57	<0.001	3.76	2.59	<0.001
HGI breakfast	4.62	4.24					34.76[¥#]	21.14		16.76[¥#]	8.76	
LGI breakfast	4.03	2.95					13.58	8.44		8.32	5.00	

ANOVA test has been used. Post-hoc contrasts were performed by Bonferroni test.

[¥] Significantly different ($p < 0.05$) from control conditions.

[#] Significant difference ($p < 0.05$) between HGI and LGI breakfasts.

HGI: High glycemic index; LGI: Low glycemic index.
