# Supplementary material for: Postprandial Effects of Breakfast Glycemic Index on Vascular Function among Young Healthy Adults: A Crossover Clinical Trial
Source: Nutrients. 2017 Jul 7;9(7):712. doi: 10.3390/nu9070712 (PMC5537827; doi:10.3390/nu9070712)
Supplement: Supplementary file 1 [file nutrients-09-00712-s001.zip › Table S3.pdf]

Table S3: Effects of type of breakfast on postprandial responses

Source			Type III Sum of Squares	Mean Square	F	Sig.	Partial Eta Squared	Observed Power
**AIx * breakfast**	Global	Sphericity Assumed	1411	235	3.4	.003	.055	0.94
		Greenhouse-Geisser	1411	252	3.4	.004	.055	0.93
	Males	Sphericity Assumed	1752	292	3.2	.005	.101	0.92
		Greenhouse-Geisser	1752	331	3.2	.008	.101	0.89
	Females	Sphericity Assumed	221	37	0.8	.601	.026	0.30
		Greenhouse-Geisser	221	40	0.8	.592	.026	0.29
**AP * breakfast**	Global	Sphericity Assumed	188	31	3.2	.005	.051	0.92
		Greenhouse-Geisser	188	35	3.2	.007	.051	0.90
	Males	Sphericity Assumed	208	35	2.9	.010	.092	0.89
		Greenhouse-Geisser	208	41	2.9	.015	.092	0.84
	Females	Sphericity Assumed	36	6	0.7	.622	.025	0.29
		Greenhouse-Geisser	36	7	0.7	.606	.025	0.27
**HR * breakfast**	Global	Sphericity Assumed	595	99	7.2	.000	.110	1.00
		Greenhouse-Geisser	595	106	7.2	.000	.110	1.00
	Males	Sphericity Assumed	230	38	2.7	.016	.086	0.86
		Greenhouse-Geisser	230	48	2.7	.026	.086	0.79
	Females	Sphericity Assumed	426	71	5.3	.000	.156	1.00
		Greenhouse-Geisser	426	72	5.3	.000	.156	0.99
**PPP * breakfast**	Global	Sphericity Assumed	611	102	2.6	.016	.043	0.86
		Greenhouse-Geisser	611	116	2.6	.021	.043	0.82
	Males	Sphericity Assumed	397	66	1.5	.174	.051	0.58
		Greenhouse-Geisser	397	82	1.5	.189	.051	0.51
	Females	Sphericity Assumed	302	50	1.4	.204	.048	0.55
		Greenhouse-Geisser	302	54	1.4	.209	.048	0.53

**CPP * breakfast**	Global	Sphericity Assumed	332	55	2.0	.070	.033	0.72
		Greenhouse-Geisser	332	63	2.0	.079	.033	0.68
	Males	Sphericity Assumed	181	30	1.2	.289	.042	0.48
		Greenhouse-Geisser	181	37	1.2	.295	.042	0.42
	Females	Sphericity Assumed	206	34	1.0	.396	.035	0.41
		Greenhouse-Geisser	206	49	1.0	.387	.035	0.33
**Glucose * breakfast**	Global	Sphericity Assumed	1727	432	3.5	.009	.056	0.86
		Greenhouse-Geisser	1727	506	3.5	.013	.056	0.81
	Males	Sphericity Assumed	330	82	1.0	.433	.033	0.30
		Greenhouse-Geisser	330	110	1.0	.416	.033	0.25
	Females	Sphericity Assumed	2024	506	3.1	.018	.099	0.80
		Greenhouse-Geisser	2024	599	3.1	.025	.099	0.75
**Insulin * breakfast**	Global	Sphericity Assumed	9554	2389	31.5	.000	.350	1.00
		Greenhouse-Geisser	9554	3750	31.5	.000	.350	1.00
	Males	Sphericity Assumed	4538	1135	10.9	.000	.277	1.00
		Greenhouse-Geisser	4538	1880	10.9	.000	.277	1.00
	Females	Sphericity Assumed	5072	1268	24.9	.000	.466	1.00
		Greenhouse-Geisser	5072	1846	24.9	.000	.466	1.00

Repeated measures ANOVA using the general lineal model (GLM) has been performed.

AIx: augmentation index; AP: augmentation pressure; HR: heart rate; PPP: peripheral pulse pressure; CPP: central pulse pressure.
