# Supplementary figures and images for: Postprandial Effects of Breakfast Glycemic Index on Vascular Function among Young Healthy Adults: A Crossover Clinical Trial
Source: Nutrients. 2017 Jul 7;9(7):712. doi: 10.3390/nu9070712 (PMC5537827; doi:10.3390/nu9070712)

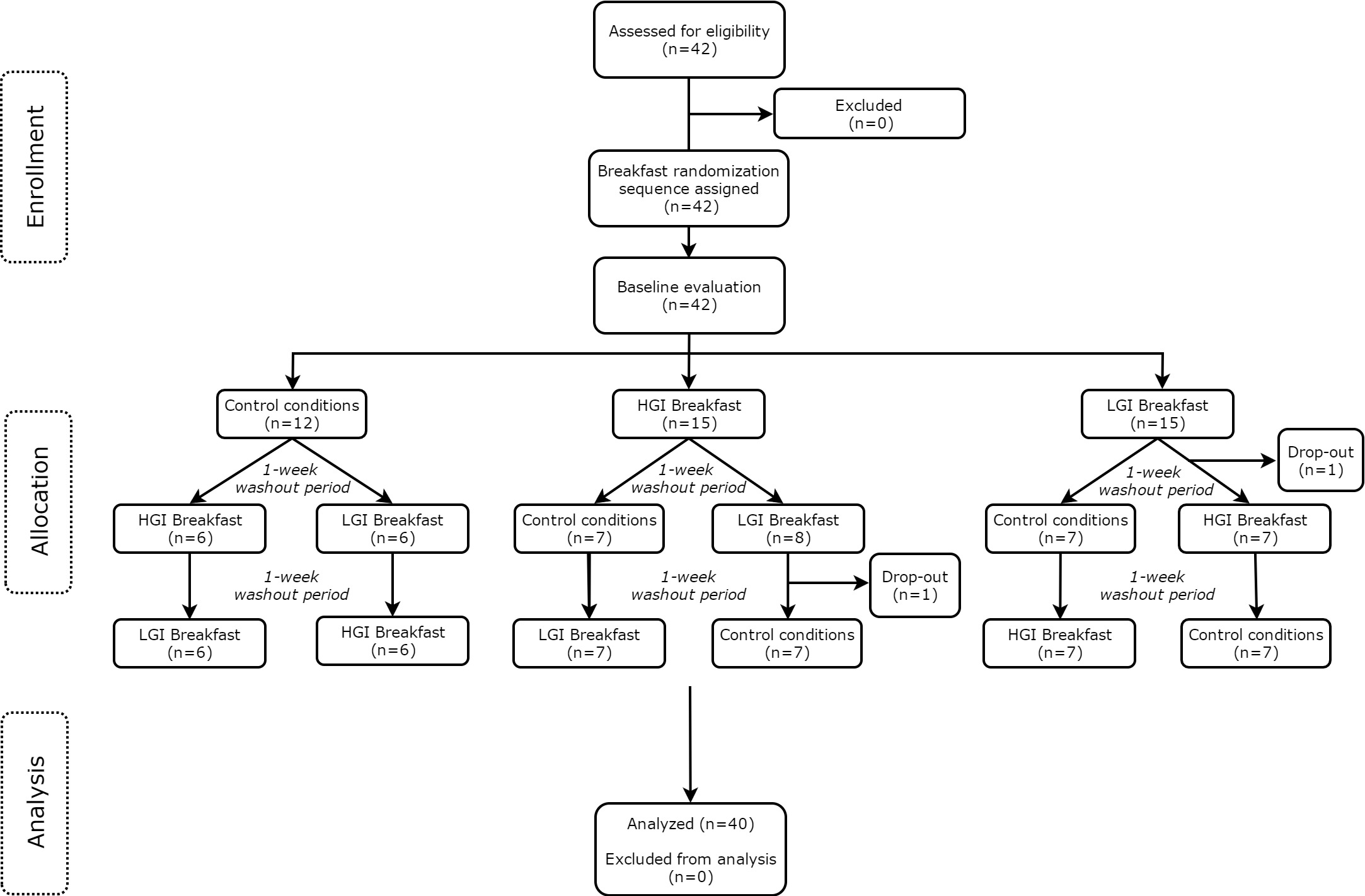

Supplement: Supplementary file 1 [file nutrients-09-00712-s001.zip › FlG S1.tif]
